# Entomophagy and chemical element residues: Noncarcinogenic risk assessment for human consumption

**DOI:** 10.1371/journal.pgph.0003462

**Published:** 2025-04-25

**Authors:** Chigozie Damian Ezeonyejiaku, Charles Obinwanne Okoye, Daniel Ogbonnaya, Nonye Juliana Ezeonyejiaku, Innocent Ikechukwu Offorbuike, Kingsley Chukwuebuka Okoye

**Affiliations:** 1 Department of Zoology, Nnamdi Azikiwe University Awka, Awka, Anambra State, Nigeria; 2 Department of Zoology & Environmental Biology, University of Nigeria, Nsukka, Nigeria; 3 Biofuels Institute, School of Environment & Safety Engineering, Jiangsu University, Zhenjiang, China; 4 Anambra State Ministry of Health, Awka, Anambra State, Nigeria; University at Buffalo, UNITED STATES OF AMERICA

## Abstract

Entomophagy has received considerable attention due to its vulnerability to toxic chemical elements, thus requiring safety assessment. In this study, edible insects *Rhyncophorus phoenics* larvae (palm weevil) and *Macrotermes bellicosus* (termite) commonly found and sold in Anambra State, Nigeria, were collected from market vendors and identified. The concentration of chemical elements was determined by Varian AA240 Atomic Absorption Spectrometer, and levels were compared with recommended standards. Noncarcinogenic risk assessment via oral exposure was evaluated following the procedure of the United State Environmental Protection Agency (USEPA). In palm weevils, zinc (Zn) had the highest concentration, whereas manganese (Mn) concentration was highest in termites. Both insects had arsenic (As) as the lowest chemical element measured, indicating low metalloid contamination. There was no difference (*P *> 0.05) compared with standard permissible daily intakes. Noncarcinogenic risk assessment showed no potential risk to consumers, demonstrating that, under the conditions of the current study, public health risk appears negligible.

## 1. Introduction

The amount of toxic chemical elements in the environment has increased in recent times as a result of human activities [1–3]. These human activities include increased application of fertilisers, use of insecticides and pesticides, industrialization, and urbanization [4,5]. Given the rising demand for animal protein, edible insects such as African palm weevils and termites seem to be a very important source of food rich in protein, iron, and other micronutrients to humans [6–8].

Notwithstanding the dietary and health advantages, it is vital to draw attention to the potential risk (as a result of chemical element contamination) of eating edible insects. This is because edible insects may be gathered from the wild or different agro-ecosystem and may be prepared for consumption in various ways, including steaming, roasting, smoking, frying, stewing, and curing, among others which may have exposed them to metal and metalloid contamination. Also, it has been established that edible insect such as termite species accumulates heavy metals due to their role as agents of soil denudation, as well as during the construction of their nests [9]. Also, owing to the habitat and feeding ecology of edible insects such as *Rhynchophorus phoenicis,* commonly known as African palm weevil, they may be exposed to various contaminants in their habitat. The potential of bioaccumulation and trophic biotransference of chemical elements in insects and the risk of public health impact on consumers are high and concerning [10]. Overall, these aspects can represent a food safety concern for edible insect consumers and should continue to be a priority for scientists.

The toxicity of chemical elements can damage organs or tissue depending on concentration and levels. Even in relatively small concentrations, some metals can be extremely hazardous [11]. While some elements such as Zn, Cu, Fe, and Mn are considered essential for biological functions, supporting enzymatic activity and overall metabolism (but only when present in small amounts), others like Cd, As, Hg, and Pb are non-essential and toxic, posing potential health risks even at low concentrations. These toxic elements do not serve any known beneficial role in biological systems and their accumulation can result in severe health consequences [12]. However, As, Hg, Ni and Pb are sometimes referred to as toxic elements and have been linked to adverse reproductive outcomes, neurological disorders, and impaired cognitive development in adults and children [13]. Also, Cd and Hg are hazardous even in low quantities and serve no useful purpose in the bodies of living things [14].

Given these concerns, it is crucial to assess the risks associated with consuming insects that may accumulate these chemical elements. Besides, the increasing consumption and demand for these insects in Anambra State, Nigeria, has made it necessary to assess risks associated with consuming African palm weevil *R. phoenicis* larvae and *Macrotermes bellicosus* (winged termite), which serves as a food alternative and are often exposed to varying concentrations of chemical elements in their natural environments. Therefore, this study was aimed to assess the noncarcinogenic risk of edible insects *R. phoenics* larvae and *M. bellicosus* commonly sold and consumed in Anambra State, Nigeria, exposed to different concentrations of chemical elements.

## 2. Materials and methods

### 2.1. Location description

**Fig 1:**
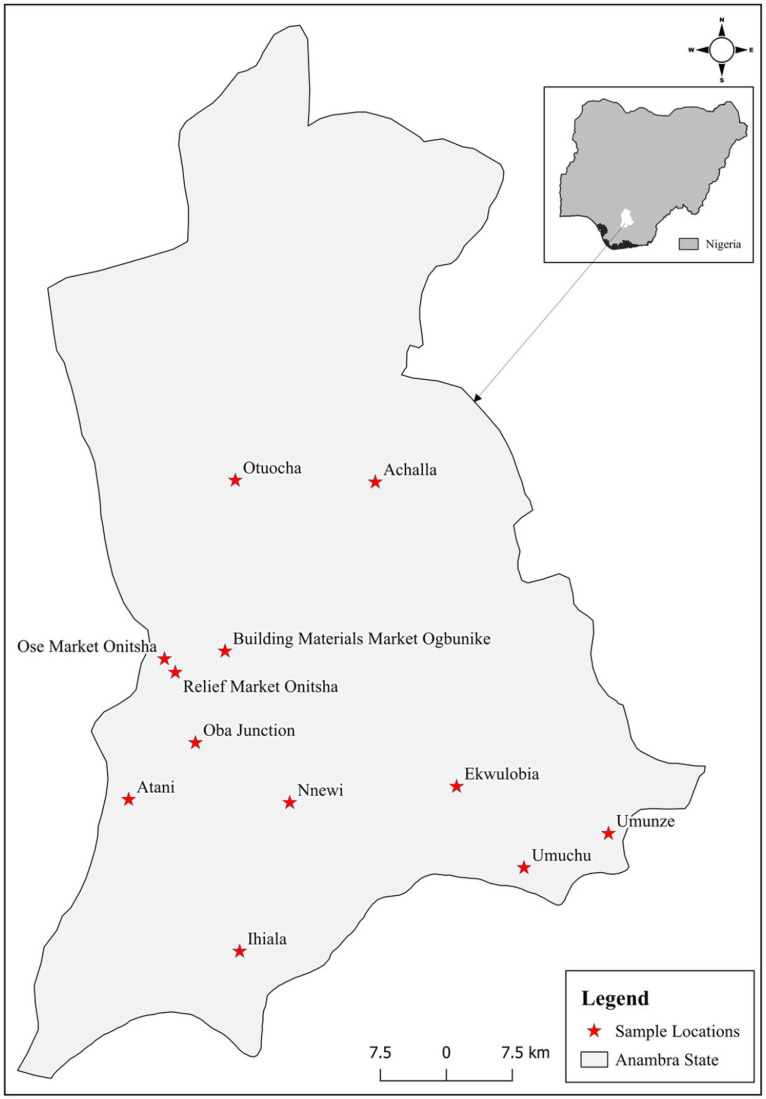
Map of Anambra State showing sample locations. The map was created using QGIS software (version 3.34, QGIS Development Team, 2023) with shapefiles obtained from Natural Earth Data (naturalearthdata.com, accessed February 2025). Sampling locations are marked with red dots.

Twelve sites, Relief market, Ose market, Oba junction, Umuchu, Umunze, Ihiala, building materials Ogbunike, Achalla, Awka, Nnewi, Atani, and Otuocha were selected for the study (**Fig 1**). The insects were collected from the market vendors in these selected sample locations. The choice of these locations was because of the presence of the insect in their markets. The sample points are the agricultural and industrialized areas of Anambra State which have their share of chemical element contamination due to varying degrees of activities in the areas [15].

### 2.2. Sampling, preparation, digestion, and analysis

The African palm weevil *R. phoenicis* and *M. bellicosus* were collected from the market vendors in the sampled sites (in their ready-for-consumption form). One hundred and forty-four *R. phoenicis* larvae were collected, and two hundred and four *M. bellicosus* was also collected and used for the study. The collected insects were placed in labelled vials that contained about 70% ethanol for preservation. The collected insects were identified using their morphological features. Prior to chemical analysis, the insects were subjected to a digestion process to prepare them for elemental analysis. Each sample was digested using concentrated nitric acid (HNO₃) and perchloric acid (HClO₄) in a ratio of 4:1. The samples were placed in digestion tubes, heated gradually to 180°C until the solution became clear, and then allowed to cool. After digestion, the resulting solution was diluted with deionized water up to a final volume of 50 mL. This acid digestion procedure ensures that all organic matter is fully dissolved, allowing for the accurate quantification of trace metal concentrations in the samples.

Chemical element analysis was conducted using Varian AA240 Atomic Absorption Spectrometer (FAAS; SpectrA 240B Agilent technologies, Victoria, Australia) according to the method of American Public Health Association (APHA) [16]. A series of standard metal solutions in the optimum concentration range was prepared, and the reference solutions were prepared daily by diluting the single stock element solutions with water containing 1.5 ml concentrated nitric acid/litre. A calibration blank was prepared using all the reagents except for the metal stock solutions. A calibration curve for each metal was prepared by plotting the absorbance of standards versus their concentrations (S1 and S2 Tables). The limit of detection (LOD) and limit of quantification (LOQ) of these chemical elements, which are crucial in determining the sensitivity of the analytical method employed was calculated using the formulas LOD = 3 σ/S, LOQ = 10 σ/S respectively where σ is SD (standard deviation) of the y-intercept (absorbance) of linear regression and S is the slope of the calibration curve (absorbance vs. concentration). During chemical element analysis, precautions were followed to prevent cross-contamination. Quality assurance and control methods were carefully practised ensuring accurate procedures. The accuracy of the analytical methods was assessed by analysing the Certified Reference Materials NIST 1640 (water matrix). Each analytical batch of ten runs was accompanied by an acid blank and three certified reference materials (CRM). Mean recoveries were in an acceptable range (90–99.5%) compared to the CRM theoretical or certified values for the chemical elements described by Ezeonyejiaku and Obiakor [17]. Results were expressed in mg kg^−1^ dry weight with a detection limit of 0.001 mg kg^−1^ dry weight and compared with FAO/WHO Codex Alimentarius (or Food Code). The Codex has worked since 1963 to create harmonised international food standards to protect consumers’ health and ensure fair trade practices [18].

Data normality was checked using the Shapiro–Wilk test, while Levene’s test was used to analyse homogeneity of variance. One-way analysis of variance was used to compare differences in chemical element concentrations between locations and test for location effect. Also, a parametric one-sample Student’s t test was used to compare the concentrations of chemical elements with FAO/WHO standards for normally distributed data.

### 2.3. *Exposure and noncarcinogenic risk assessment*

The human exposure pathway of heavy metals through the consumption of *R. phoenicis* larvae and *M. bellicosus* were evaluated using USEPA model [18,19]. To characterise oral metal exposure and chronic noncarcinogenic risk, the target hazard quotient (THQ) was calculated using accepted techniques and presumptions [20].


$$\mathrm{\backslash [}THQ=\frac{ADI}{RfD}\;where\;ADI=\frac{MC\;\times \;IR\;\times \;EF\;\times \;ED\;\times \;CF}{BW\;\times \;ATn}\;is\;the\;average\;daily\;intake\mathrm{\backslash ]}$$

MC = metal mean concentration (mg kg^−1^), IR = the daily ingestion rate (adult) (0.04 kg), EF = the exposure frequency (365 days/year), ED = the exposure duration for noncarcinogens (30 years), CF = the conversion factor (0.208) at 79% of moisture content, BW = average adult body weight (70 kg), ATn = average exposure time for noncarcinogens (10,950 days), and RfD = reference dose of individual metals, including lead, cadmium, copper, zinc, iron, manganese, nickel, mercury, chromium and arsenic at 3.5E−03, 1.00E−03, 4.00E−01, 3.00E−01, 7.00E−01, 1.40E−01, 2.00E−02, 1.60E−04, 3.00E−03 and 3.00E−04 mg kg^−1^ day− 1, respectively) (Ezeonyejiaku et al., 2020). The hazard index (HI) was further calculated from THQ values as follows: HI = THQ (As) + THQ (Pb) + THQ (Cr) + THQ (Cd) + THQ (Mn) + THQ (Hg) + THQ (Ni) + THQ (Cu) + THQ (Zn) + THQ (Fe) [21].

## 3. Results and discussion

### 3.1. Concentration of chemical elements in palm weevil (larvae) and termites

The mean and standard deviation of metal concentration in *R. phoenicis* and *M. bellicosus* collected from the sample sites, and comparison with FAO/WHO standard are illustrated in **Table 1**. There were observed differential patterns in the accumulation of the sampled essential and non-essential chemical elements, which may be linked to species variation, life stages, environmental factors, feeding biology, and trophic ecology. The mean values for the metals in *R. phoenicis* are in the decreasing order of Zn> Hg> Pb> Mn> Cd> Ni> Cr> Fe> Cu>As. While the concentration of the metals in *M. bellicosus* is in the decreasing order of Mn>Zn> Fe> Cu> Hg> Pb>Cd>Cr>Ni and As. The study showed that *R. phoenicis* larvae accumulated more Zinc (Zn), while *M. bellicosus* accumulated more Manganese (Mn) compared to other chemical elements. However, Arsenic (As) was not detected in both species.

**Table 1:** Mean concentration of chemical elements in *R. phoenicis* larvae and *M. bellicosus.*

Chemical elements	*R. phoenicis* (mg kg^−1^)	*M. bellicosus* (mg kg^−1^)	LOD*	LOQ**	FAO/WHO (mg kg^−1^)
***Essential***					
Copper (Cu)	0.002 ± 0.003	0.185 ± 0.007	0.0006	0.0014	30
Zinc (Zn)	1.750 ± 0.070	1.46 ± 0.004	0.0005	0.0007	30
Iron (Fe)	0.008 ± 0.001	0.332 ± 0.421	0.0009	0.0016	–
Manganese (Mn)	0.064 ± 0.015	7.508 ± 0.700	0.0006	0.0012	–
Nickel (Ni)	0.03 ± 0.004	0.008 ± 0.001	0.0008	0.0019	–
***Non-essential***					
Lead (Pb)	0.133 ± 0.013	0.112 ± 0.311	0.0014	0.0025	1–6
Cadmium (Cd)	0.031 ± 0.006	0.375 ± 0.021	0.0010	0.0018	2.0
Mercury (Hg)	0.375 ± 0.125	0.155 ± 0.026	0.0003	0.0010	1.0
Chromium (Cr)	0.008 ± 0.001	0.016 ± 0.001	0.0012	0.0035	1.0
Arsenic (As)	ND	ND	0.0004	0.0020	1–3.5

* Limit of Detection, **Limit of Quantification. *Values are mean ± standard deviation of tri-replicates. ND not detected, FAO/WHO Food and Agriculture Organization and World Health Organization.*

Owing to the increase in the consumption of edible insects worldwide, the need for food safety issues is a concern, particularly in developing countries after the recognition of insects as food which has seen a slight increase in their consumption [18]. Limited knowledge of food safety as it relates to edible insects in many countries is a barrier to promoting edible insect farming and consumption in some niches. According to Rochow et al. [22] and Murefu et al. [23], the food safety of edible insects needs to be investigated, particularly in sub-Saharan African countries in order to encourage and promote the use of insects in human diets. The knowledge of rearing and using insects in food production, including consumption as food ingredients, is not well known [21]. Apart from the nutritional benefits associated with edible insects, they constitute exogenous and endogenous risk factors to human health, as in the case of animal and plant-based foods.

The mean copper (Cu) values of 0.002 mg kg^−1^ and 0.185 mg kg^−1^ found in *R. phoenicis* larvae and *M. bellicosus*, fall within the FAO/WHO recommended value for copper [18]. The value obtained in the study were lower than that obtained by Abdullahi et al. [21] and Ezeonyejiaku et al. [15] with 0.13–0.86 mg kg^−1^ and 18.56 ± 0.01 - 78.02 ± 2.35 mg kg^−1^ respectively. Copper which is an essential element in the body and is needed by every adult human daily to help enzymes transfer energy in cells. Despite being a necessary micronutrient for the human body’s synthesis of blood, copper can have negative effects on the kidney, liver, stomach, and anaemia when consumed in amounts over the recommended dose.

When present in tiny amounts, one of the nutrients needed by the human body for healthy functioning is zinc [24]. The zinc (Zn) concentration for *R. phoenicis* larvae and *M. bellicosus* recorded a mean value of 1.750 mg kg^−1^ and 1.46 mg kg^−1^, respectively, thus, these values are way below the recommended value by FAO/WHO (2020). The findings of the study were comparable to the result obtained in the study of Ezeonyejiaku et al. [15]. However, the result of the study was significantly lower than that obtained by Abdullahi et al. [21] in grasshopper 281.48 ± 45.52 mg kg^−1^, locust 274.74 ± 79.39 mg kg^−1^, termite 259.34 ± 16.10 mg kg^−1^. Zinc is essential for all living things, forming the active site in over 20 metallo-enzymes. It can be carcinogenic in excess. According to Kumar and Mukheerje [25], a divalent form of zinc is the second most abundant element in the human body and modulates the activity of protein folding and function.

The concentrations of iron (Fe) recorded in this study were 0.008 mg kg^−1^ for *R. phoenicis* and 0.332 mg kg^−1^ for *M. bellicosus,* respectively. Though no FAO/WHO limit exists for Fe, the values obtained were significantly lower than that obtained by Abdullahi et al. [21] and Ajai et al. [26]. Iron is an essential element for all forms of life, it is a component of haemoglobin and many enzyme systems. As a component of the haemoglobin molecule in red blood cells, iron is a vital element that the body needs for oxygen transport. However, when levels of iron are in excess, free radicals may develop and cause liver damage [18]. The concentrations of manganese found in *R. phoenicis* larvae and *M. bellicosus* were 0.064 mg kg^−1^ and 7.508 mg kg^−1^, respectively. Though no FAO/WHO limit exists for Mn, studies indicate that dietary manganese intakes range from 1–2 mg/day in mild hospital diets to around 18 mg/day for diets consisting predominantly of vegetables, nuts, and seeds [27] (USEPA, 1995). These values are higher the values recorded in this study.

The Mean value of *R. phoenicis* and *M. bellicosus* for Nickel were 0.03 mg kg^−1^ −0.008 mg kg^−1^, respectively, though no FAO/WHO limit exists for Ni, it is a necessary element for both animal and human health [28]. High concentrations of nickel can produce allergic reactions and harm the immune system, kidneys, heart, and reproduction.

The lead (Pb) concentration was 0.133 mg kg^−1^ and 0.112 mg kg^−1^ in *R. phoenicis* and *M. bellicosus,* respectively, and was below the recommended standard of 1–6 mg kg^−1^ for food resources [18]. On the other hand, the value obtained was also lower than those reported by Ezeonyejiaku et al. [15] and Abdullahi et al. [21] (0.14–4.97 mg kg^−1^ and 1.14 ± 0.49 to 0.04 ± 0.08 mg kg^−1^ respectively). The role of Lead (Pb) in the body’s healthy functioning is unknown. It is toxic and carcinogenic and, if accumulated in the body, can cause serious health challenges like disturbance of the development of an embryo or foetus.

The concentration of cadmium recorded was 0.031 mg kg^−1^ for *R. phoenicis* and 0.037 mg kg^−1^ for *M. bellicosus*. These values were below the recommended permissible limit of 2.0 mg kg^−1^ set by FAO/WHO [18]. The concentration of cadmium in the study was lower that the value reported by Abdullahi et al. [21], which ranges from 1.74 ± 0.00 to 7.11 ± 0.89 mg kg^−1^. However, the value was similar to the result reported by Ezeonyejiaku et al. [15], which was 0.02–0.19 mg kg^−1^, respectively. These variations in the concentration may be due to the habitat and feeding mode of the insects [26]. Food is the main source of daily exposure to cadmium. On average, some milligrams are taken in per day, and it accumulates in the body. A normal individual absorbs about 6% of ingested cadmium, but up to 9% may be absorbed by those with iron deficiency [18].

The mercury concentration was 0.375 mg kg^−1^ −0.155 mg kg^−1^ for *R. phoenicis* and *M. bellicosus,* respectively. Thus, these values found in the edible insects were below the recommended FAO/WHO limits of 1.0 mg kg^−1^. The results of the study were lower than the values obtained in the study of Ezeonyejiaku et al. [15]. Also, the result of the study was significantly lower than that obtained by Abdullahi et al. [21]. Hg is a naturally occurring pollutant, and its elevated concentration is frequently accelerated by industrial operations and emissions, with concomitant public health consequences such as neurobehavioral and developmental diseases. The concentration of chromium was 0.008 mg kg^−1^ −0.016 mg kg^−1^ for *R. phoenicis* and *M. bellicosus,* respectively. These values found in our study were below the recommended FAO/WHO limits of 1.0 mg kg^−1^. Studies have shown the nutritional importance of low Cr levels as an essential metal for human consumption [29]. The result of the study shows that As was not detected in the two insect species studied. However, while the concentration of Hg and As, was determined using the AAS, it is important to recognize that standard AAS may not be the most suitable method for accurate analysis of Hg and As, especially at minimal or low concentrations. More advanced methods, such as Hydride Generation Atomic Absorption Spectrophotometry (HGAAS) for As and Cold Vapor Atomic Absorption Spectrophotometry (CVAAS) for Hg, are specifically designed to handle the volatility of these elements and provide more accurate results. These methods are recommended for future studies to ensure precise quantification of Hg and As at low levels.

### 3.2. Risk assessment of chemical elements in palm weevil (larvae) and termites

A target hazard quotient (THQ)-based public health risk assessment was carried out using the US EPA’s tolerable value of 1 [30]. **Table 2** displays the estimated THQs for lead, cadmium, copper, zinc, iron, manganese, nickel, mercury, chromium, and arsenic found in *R. phoenicis* and *M. bellicosus*. The THQ estimations ranged from ND- 2.7E-01 for all metals and were below 1. The two insects (*R. phoenicis* and *M. bellicosus*) with hazard index (HI) (1.8E-01 to 2.9E-01) was below 1, which indicates that oral exposure by consumption of *R. phoenicis* and *M. bellicosus* with heavy metal was not linked with any potential noncarcinogenic danger to public health [31].

**Table 2:** Target Hazard Quotient (THQ) and Hazard Index (HI) of chemical elements in *R. phoenicis* larvae and *M. bellicosus.*

Chemical element		THQ
	***R. phoenicis***	***M. bellicosus***
***Essential***		
Cu	5.94E-07	5.5E-05
Zn	6.9E-03	5.7E-03
Fe	1.36E-06	5.64E-05
Mn	5.43E-05	6.3E-03
Ni	1.7E-03	4.7E-04
***Non-essential***		
Pb	4.5E-04	3.8E-04
Cd	3.6E-03	4.4E-02
Hg	2.7E-01	1.1E-01
Cr	3.1E-03	6.3E-03
As	NC	NC
**HI**	**2.9E-01**	**1.8E-01**

NC; not calculated as metals since they were not detected in *Rhynchophorus phoenicis* larvae and *Macrotermes bellicosus*, Pb Lead, Cd Cadmium, Cu Copper, Zn Zinc, Fe Iron, Mn Manganese, Ni Nickel, Mercury Hg, Chromium Cr, Arsenic As, THQ Target Hazard Quotient, HI hazard index.

### Limitations of the study

Some limitations were encountered in the present study. First, while the results suggest that these insects are safe for human consumption under current conditions, the lack of detailed sourcing information regarding the exact locations from which the insects were harvested is a limitation, as environmental factors may influence the levels of chemical elements. Additionally, the use of CRM (NIST 1640, water matrix) for quality control, which is designed for aqueous samples, may introduce accuracy issues due to matrix differences when applied to solid biological samples. Although thorough digestion was ensured and the method validated for solid samples, elements with varying behaviors could still be affected. Future studies should address these limitations by tracing insect sample origins, evaluating the impact of both processing methods and environmental conditions, and using CRMs specifically tailored to digested biological matrices. Furthermore, continuous monitoring of additional chemical elements and toxic pollutants in edible insects is crucial for promoting their safe consumption.

## 4. Conclusion

This study examined the level of chemical elements in two edible insects in twelve sites (Relief market, Ose market, Oba junction, Umuchu, Umunze, Ihialla, Building materials Ogbunike, Achalla, Awka, Nnewi, Atani and Otuocha) in Anambra State and its public health risk assessment. Cu, Zn, Fe, Mn, Ni, Cr, As, Pb, Cd, and Hg were below the tolerable limits. However, the concentrations of chemical elements found in the two edible insects were in the order of *R. phoenicis *< *M. bellicosus*. Furthermore, the THQ and HI values for these chemical elements were less than 1, indicating that these insects are acceptable for human consumption with no health concerns present in the specified area of the study. Although the THQ and HI calculations confirmed no quantitative estimate of public health risks from the consumption of these insect species, it has provided information on the safety of edible insect consumption in Anambra State.

## Supporting information

S1 Table Standard calibration curve for nitrate determination. (DOCX)

S2 Table Percentage recovery of metals. (DOCX)
