# Supplementary material for: Entomophagy and chemical element residues: Noncarcinogenic risk assessment for human consumption
Source: PLOS Glob Public Health. 2025 Apr 25;5(4):e0003462. doi: 10.1371/journal.pgph.0003462 (PMC12027056; doi:10.1371/journal.pgph.0003462)
Supplement: S1 Table — (DOCX) [file pgph.0003462.s001.docx]

**S1 Table: Standard Calibration Curve for Nitrate Determination**

| **Ml of Standard Solution** | **Ml of Distilled water** | **Concentration (mg/l)** | **Absorbance** |
| --- | --- | --- | --- |
| 0.00 | 1.00 | 0.00 | 0.00 |
| 0.20 | 0.80 | 0.02 | 0.04 |
| 0.40 | 0.60 | 0.04 | 0.12 |
| 0.60 | 0.40 | 0.06 | 0.17 |
| 0.80 | 0.20 | 0.08 | 0.20 |
| 1.00 | 0.00 | 0.10 | 0.26 |
