# Supplementary material for: Entomophagy and chemical element residues: Noncarcinogenic risk assessment for human consumption
Source: PLOS Glob Public Health. 2025 Apr 25;5(4):e0003462. doi: 10.1371/journal.pgph.0003462 (PMC12027056; doi:10.1371/journal.pgph.0003462)
Supplement: S2 Table — (DOCX) [file pgph.0003462.s002.docx]

**S2 Table: Percentage recovery of metals.**

| Samples | Cr ppm | | As ppm | | Cu ppm | | Hg ppm | | Zn ppm | | Lead ppm | | Mn ppm | |
| --- | --- | --- | --- | --- | --- | --- | --- | --- | --- | --- | --- | --- | --- | --- |
|  | 1^st^ | 2^nd^ | 1^st^ | 2^nd^ | 1^st^ | 2^nd^ | 1^st^ | 2^nd^ | 1^st^ | 2^nd^ | 1^st^ | 2^nd^ | 1^st^ | 2^nd^ |
| Spike sample ppm | 9.07 | 9.07 | 9.09 | 9.08 | 10.89 | 10.93 | 9.07 | 9.07 | 11.89 | 11.11 | 9.28 | 9.47 | 10.67 | 10.72 |
| Unspike sample ppm | 0.6 | 0.72 | 0.24 | 0.29 | 1.98 | 1.87 | 0.22 | 0.25 | 2.76 | 2.70 | 0.298 | 0.280 | 0.987 | 0.955 |
| Original conc spiked ppm | 10.00 | 10.00 | 10.00 | 10.00 | 10.00 | 10.00 | 10.00 | 10.00 | 10.00 | 10.00 | 10.00 | 10.00 | 10.00 | 10.00 |
| % recovery | 84.7 | 83.5 | 88.5 | 87.9 | 89.1 | 90.6 | 88.5 | 88.2 | 91.3 | 84.1 | 89.82 | 91.9 | 96.83 | 97.65 |
